# CT-guided Lumbar Puncture for Intrathecal Nusinersen Injection in Patients with Spinal Muscular Atrophy: Technical Effectiveness, Safety, and Radiation Dose

**DOI:** 10.1007/s00062-025-01590-9

**Published:** 2025-11-25

**Authors:** Yannick Laurent Thal, Marcel Opitz, Raya Ocker-Serger, Laura Valentina Klüner, Daniel Rosok, Marcel Alexander Drews, Denise Bos, Johannes Haubold, Christoph Kleinschnitz, Tim Hagenacker, Michael Forsting, Cornelius Deuschl, Sebastian Zensen

**Affiliations:** 1 Essen University Hospital, Essen, Germany; 2 University Hospital of Zurich, Zurich, Switzerland

**Keywords:** Spinal muscular atrophy (SMA), Nusinersen, Intrathecal drug administration, CT-guided lumbar puncture, Radiation exposure, Dose optimization

## Abstract

**Purpose:**

Spinal muscular atrophy (SMA) is a rare neuromuscular disease treated with intrathecal nusinersen. In patients with complex spinal anatomy or spinal instrumentation, repeated lumbar punctures can be challenging. This study evaluated the feasibility, safety, and radiation exposure of CT-guided nusinersen administration in SMA.

**Methods:**

In this retrospective single-center study, 458 CT-guided nusinersen injections in 44 SMA patients (October 2017–August 2024) were analyzed. Technical success, complications, procedure and puncture times, and radiation exposure were compared between subgroups. Procedures were performed in prone, lateral, or combined (oblique) positions depending on anatomy.

**Results:**

Technical success was 98.3% (449/458). Rates were slightly lower in patients with dorsal spondylodesis (97.1% vs. 98.8%). Complications occurred in 1.1% (5/458), including transient pain and two hematomas. Spondylodesis was associated with longer puncture (11.9 vs. 10.3 min) and procedure durations (16.3 vs. 14.0 min) and higher radiation doses (effective dose 2.22 vs. 1.74 mSv; all *p* < 0.01). Failed or complicated procedures showed prolonged durations and higher exposure. Needle repositioning correlated with duration and dose.

**Conclusion:**

CT-guided lumbar puncture for intrathecal nusinersen injection is a safe and effective technique, even in complex spinal anatomy. Dorsal spondylodesis increases procedural complexity. Tailored puncture level selection, optimized positioning, and dose-reduction strategies are essential for high success rates and minimal radiation. While CT guidance ensures excellent anatomical visualization, fluoroscopy may reduce radiation and procedure time; future comparative studies should define optimal modality selection.

## Introduction

Spinal muscular atrophy (SMA) is a rare autosomal recessive motor neuron disease characterized by progressive degeneration of motor neurons in the anterior horn of the spinal cord [1, 2]. Clinically, it manifests with generalized muscle hypotonia and atrophy, which can present from early infancy to adulthood, depending on the disease subtype [3, 4]. The underlying cause is a mutation in the *SMN1* gene, while disease severity correlates with the number of *SMN2* gene copies [5–7]. The SMN1 gene encodes the survival motor neuron (SMN) protein, which is essential for the maintenance of motor neurons and for spliceosome assembly in RNA processing. Its paralog SMN2 produces only small amounts of functional SMN protein due to alternative splicing that excludes exon 7, and therefore partially compensates for the loss of SMN1. Clinically, SMA is divided into subtypes based on age of onset and maximal motor function: type 1 (Werdnig–Hoffmann disease) presents in infancy with severe hypotonia and inability to sit unsupported; type 2 manifests in early childhood with the ability to sit but not walk independently; and type 3 (Kugelberg–Welander disease) has later onset and milder progression, with patients initially able to walk.

With the approval of nusinersen in 2017, the first disease-modifying therapy became available. It enhances the alternative splicing of *SMN2* transcripts, thereby increasing production of functional SMN protein [8, 9]. The treatment regimen consists of four loading doses administered over the first two months (at days 0, 14, 28, and 63), followed by maintenance injections every four months as lifelong therapy. The clinical benefits of nusinersen have been well demonstrated in both pediatric and adult populations, leading to widespread adoption as a standard of care [10, 11]. This is further supported by a recent systematic review and meta-analysis in adolescents and adults, which confirmed that nusinersen treatment leads to stabilization or improvement of motor function across a broad spectrum of SMA patients [12]. However, its mode of administration—repeated intrathecal injection—poses considerable logistical and technical challenges, particularly in patients with severe scoliosis, complex spinal anatomy, or prior spinal instrumentation [13–15]. Given that nusinersen is among the most expensive medications worldwide, precise and safe intrathecal administration is essential to avoid any loss of drug and to ensure full therapeutic efficacy. Image guidance such as fluoroscopy or ultrasound has been explored, but often proved insufficient in complex cases or in centers lacking specific expertise [16]. CT-guided lumbar puncture, by contrast, provides high-resolution anatomical visualization and has emerged as a safe and effective technique for intrathecal drug administration in complex cases [16, 17]. Prior case series and institutional experiences have demonstrated feasibility and safety; however, large-scale data on long-term implementation, technical success rates, and cumulative radiation exposure are still limited, especially in adult and surgically pretreated populations [13].

The aim of this study was to analyze the technical feasibility, safety profile, and radiation exposure of CT-guided intrathecal nusinersen administration in a large cohort of patients with SMA. In addition, the impact of anatomical factors—in particular dorsal spondylodesis—as well as patient positioning and puncture level were assessed.

## Material and Methods

### Study Design and Cohort

**Fig. 1:**
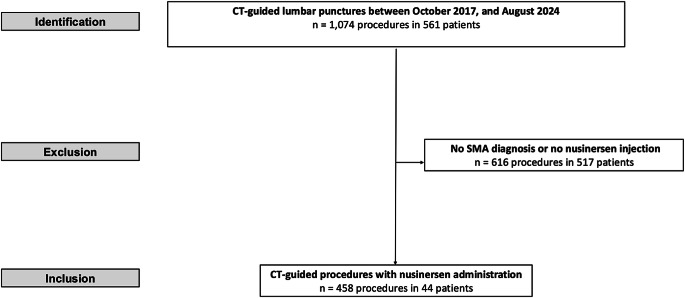
Flow chart of study cohort selection

This retrospective, single-center observational study was approved by the local ethics committee and the requirement for informed consent was waived. All CT-guided procedures performed between October 2017 and August 2024 were reviewed. The study cohort selection process is shown in Fig. 1.

### Procedural Technique

All nusinersen injections were performed under CT guidance using scanners from Siemens Healthineers: SOMATOM Definition AS+, SOMATOM Definition Flash, SOMATOM X.ceed, or NAEOTOM Alpha. CT scans were acquired in a step-and-shot technique. For image guidance, a single axial sequence was reconstructed in three slices with 3‑mm thickness, using comparable technical parameters across all scanners. The median tube voltage was 115 kV (IQR 110–120) and the median tube current time product was 87 mAs (IQR 69–128). Automatic exposure control was used in all scanners, with tube voltage reference values between 110–120 kV. All procedures were preceded by thorough preparation, including pre-procedural planning, imaging, laboratory testing, and written informed consent. Patient positioning was adapted to individual anatomical and clinical considerations, with punctures typically performed in the lateral, prone, or combined (oblique) position. The puncture level was selected based on anatomical feasibility and any prior surgical interventions (e.g., spondylodesis, sclerotic changes, osteophytes). CT-guided lumbar punctures were performed with traumatic Quincke-type spinal needles only. Two specifications were used depending on anticipated trajectory length and access complexity: 21-gauge × 3.5-inch (90 mm) Quincke needles (SPINAJECT®, Disomed GmbH & Co. KG, Gelnhausen, Germany) for standard interlaminar access, and 20-gauge × 6.00-inch (152.4 mm) Quincke needles (Becton Dickinson, Franklin Lakes, NJ, USA) for longer trajectories or increased rigidity requirements (e.g., severe scoliosis, dorsal instrumentation). Atraumatic pencil-point needles (e.g., Sprotte/Whitacre) were not used in this cohort. All punctures were performed by board-certified neuroradiologists under standardized conditions. A consistent protocol regarding antiseptic preparation and needle type was followed throughout to ensure procedural uniformity and minimize variability. Nusinersen administration was carried out by the treating neurologists. Procedural documentation, including time stamps (start, end, needle insertion), was recorded in the RIS (Radiology Information System) and PACS (Picture Archiving and Communication System). Nusinersen was administered directly through the spinal needle without the use of an intermediate infusion line. Correct intrathecal needle placement was confirmed by cerebrospinal fluid backflow. Because of the minimal dead space of the needle, no saline flush was performed after drug application. The total procedure duration was defined as the time from acquisition of the initial planning scan until completion of the intervention, including patient preparation (disinfection and sterile draping), positioning, potential repositioning, and intrathecal drug administration. The puncture duration was defined as the interval from the first scan series in which the needle was visible within the patient to successful completion of nusinersen injection. Both procedure and puncture duration shared the same endpoint at the time of completed intrathecal administration. A reposition attempt was defined as any withdrawal of the needle beyond the subcutaneous tissue followed by redirection at the same interlaminar level, using the same needle whenever feasible. A new puncture level or needle was selected only if intrathecal access could not be achieved after multiple repositioning attempts. Complications were systematically recorded both during the procedure and throughout the postprocedural hospital stay until discharge. All cases with spinal instrumentation had dorsal spondylodesis involving the lumbar spine, in some patients extending to the lower thoracic or upper sacral levels. All procedural and dose-related data were retrospectively extracted from the Radiology Information System (RIS) and Picture Archiving and Communication System (PACS) by the first author, a radiology resident with training in data analysis and image interpretation. In cases of uncertainty, the respective procedures were jointly reviewed in consensus with a board-certified neuroradiologist with over ten years of experience in spinal interventions. No formal dual reading was performed. All adverse events were evaluated based on clinical documentation and imaging findings.

### Data Collection and Evaluation

The following parameters were recorded: demographic data, SMA subtype, patient positioning, puncture level, presence of dorsal spondylodesis, technical success, number of repositioning attempts, complications during the procedure and until discharge. The total intervention time was defined as the duration from acquisition of the planning scan to the documented end of the procedure. Puncture duration was defined as the time between the first attempt at needle placement and successful intrathecal nusinersen injection. Nine procedures were excluded from time-based analysis due to incomplete documentation or procedure abortion.

### Radiation Exposure Assessment

Radiation exposure was documented for each procedure using the parameters CTDIvol (volumetric computed tomography dose index) in mGy, dose-length product (DLP) in mGy·cm, and effective dose in mSv. The effective dose for CT-guided injections was calculated according to ICRP (International Commission on Radiological Protection) Publication 103 [18]. In 11 procedures, radiation dose data were incomplete or missing and were therefore excluded from at least one dose-related analysis.

### Statistical Analysis

Statistical analysis was performed using GraphPad Prism (version 10.5.0; GraphPad Software, San Diego, CA, USA). Categorical variables (e.g., technical success, complications) were analyzed using Fisher’s exact test or Chi-squared test, as appropriate. Periprocedural continuous variables (e.g., duration and radiation parameters) were non-normally distributed and are expressed as medians with interquartile ranges (IQR). Demographic parameters such as age were analyzed descriptively. Two-group comparisons (e.g., spondylodesis vs. no spondylodesis, successful vs. failed procedures) were carried out using the Mann–Whitney U test. Comparisons across more than two groups (e.g., puncture level, patient positioning) were assessed with the Kruskal–Wallis test followed, where significant, by Dunn’s multiple comparisons post-hoc analysis. Correlations between number of needle repositioning attempts, procedural durations, and radiation dose parameters were evaluated using Spearman’s rank correlation. Statistical significance was defined as *p* < 0.05.

## Results

Out of 1074 CT-guided spinal interventions, 616 procedures in 517 patients were excluded because they were either unrelated to nusinersen injections or lacked a confirmed diagnosis of SMA (Fig. 1). The study cohort comprised 458 CT-guided procedures in 44 patients with confirmed SMA. The median age was 31 years (IQR 25–44 years, range 14–58 years). 21 (47.7%) of patients were female. SMA subtype distribution included type 1 in 6.8% (3/44), type 2 in 63.6% (28/44) and type 3 in 29.5% (13/44). Dorsal spondylodesis was present in 47.7% (21/44), corresponding to 208 of 458 procedures (45.4%). A summary of demographic and clinical characteristics is presented in Table 1.

**Table 1:** Demographic and clinical characteristics of the study cohort.

Characteristic	Value
Number of patients	44
Median Age (range)	31 years (14–58 years)
Sex	23 male (52.3%), 21 female (47.7%)
Dorsal spondylodesis present	21 patients (47.7%)
Total number of interventions performed	458
SMA type	
*Type 1*	3 patients (6.8%)
*Type 2*	28 patients (63.6%)
*Type 3*	13 patients (29.5%)

### Technical Success

**Fig. 2:**
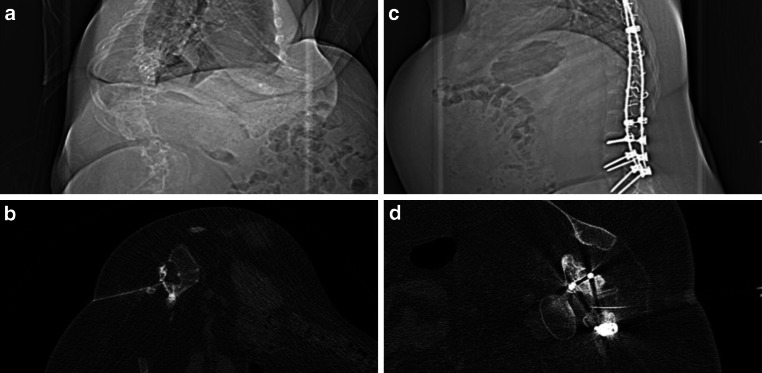
Representative CT planning images in patients with and without dorsal spinal fusion. Scout image (**a**) and corresponding axial CT image (**b**) in a patient without spinal instrumentation, showing unobstructed anatomical landmarks and a straightforward intrathecal needle path. Scout image (**c**) and axial CT image (**d**) in a patient with dorsal spondylodesis, showing increased procedural complexity due to altered bony anatomy and spinal hardware, necessitating advanced planning and angulated needle trajectory

449 out of 458 procedures were technically successful, corresponding to an overall technical success rate of 98.3%. Among patients with dorsal spondylodesis, the technical success rate was 97.1% (202/208), compared to 98.8% (247/250) in patients without dorsal spondylodesis (*p* = 0.311). A detailed comparison of technical success rates and procedure times is shown in Table 2. Notably, 66% (6/9) failed procedures occurred in patients with dorsal spondylodesis. Figure 2 illustrates the increased technical complexity of CT-guided intrathecal nusinersen administration in patients with dorsal spondylodesis compared to those without spinal instrumentation.

**Table 2:** Comparison of procedural parameters between patients with and without dorsal spondylodesis.

Parameters	With dorsal spondylodesis	Without dorsal spondylodesis	*p*-value
Number of procedures	208 (45.4%)	250 (54.6%)	–
Technically successful procedures	202 (97.1%)	247 (98.8%)	0.311
Puncture duration (median, range)	11.9 min (2.8–96.6)	10.3 min (1.6–76.0)	0.0031
Procedure duration (median, range)	16.3 min (7.4–102.9)	14.0 min (5.1–134.1)	0.0028

### Complications

Periprocedural complications occurred in 1.1% (5/458) and included 3 cases of postprocedural pain, 1 needle tract hematoma, and 1 retroperitoneal hematoma (Table 3). All complications were transient and without long-term sequelae. Complications occurred more frequently in patients with dorsal spondylodesis (4/208; 1.92%) compared to those without (1/250; 0.40%), although this difference did not reach statistical significance (*p* = 0.176). Procedures with complications or technical failure were associated with significantly prolonged procedure and puncture durations (Table 4). Post-dural puncture headache (PDPH) was not specifically recorded in the retrospective dataset, as standardized follow-up for this complication was not part of the institutional protocol at the time of data collection. However, postprocedural pain events were analyzed separately, and none were described as typical for PDPH.

**Table 3:** Overview of periprocedural complications.

Type of complication	*n*	Proportion of all interventions (%)
Pain	3	0.7
Needle tract hematoma	1	0.2
Retroperitoneal hematoma	1	0.2
All complications	5	1.1

**Table 4:** Comparison of key intervention parameters for all procedures, procedures with complications and technically unsuccessful procedures.

Parameters	All interventions (*n* = 458)	Interventions with complications (*n* = 5)	Unsuccessful interventions (*n* = 9)
Median number of repositioning attempts (IQR, range)	0 (0–0, 0–5)	1 (1–2, 0–4)	1 (0–2, 0–5)
Median puncture duration (IQR, range)	11.1 min (7.7–16.2, 1.6–96.6)	55.8 min (55.1–76, 20.9–95.7)	52.2 min (33.4–65.8, 18.4–96.6)
Median procedure duration (IQR, range)	14.8 min (10.9–20.8, 5.1–134.1)	59.7 min (59.1–81.9, 41.7–102.9)	58.4 min (45.9–69.7, 22.2–102.9)
Dorsal spondylodesis (%)	202 (44.1%)	4 (80.0%)	6 (66.7%)

### Needle Repositioning

**Fig. 3:**
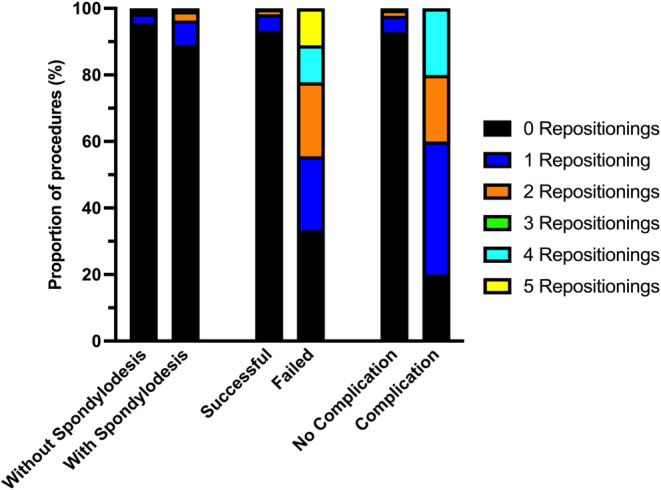
Distribution of the number of needle repositionings, stratified by the presence of dorsal spondylodesis, procedural success, and occurrence of complications. Each stacked bar represents the proportion of procedures within the respective subgroup. Most procedures were performed without needle repositioning (median 0, range 0–5). The single case requiring five needle repositionings occurred in a failed procedure without any recorded complication and is therefore visible only within the ‘Failed’ group. Each category represents an independent subgroup analysis. The figure illustrates that procedures in patients with dorsal spondylodesis, as well as failed or complicated interventions, required more frequent needle repositioning attempts

Across all 458 procedures, the median number of repositioning attempts was 0 (range 0–5). Although the median number of repositioning attempts was 0 in both groups, procedures in patients with dorsal spondylodesis required repositioning more frequently overall, resulting in a statistically significant difference compared to those without spondylodesis (*p* = 0.012). Furthermore, the five procedures with complications required significantly more repositioning attempts (median 1, range 0–4) than those without (median 0; *p* < 0.0001). In the nine technically unsuccessful procedures, the median number of repositioning attempts was 1 (range 0–5), which was significantly higher compared to successful procedures (median 0; *p* < 0.0001). Figure 3 illustrates the distribution of the number of repositionings, stratified by presence of spinal fusion, procedural success, and occurrence of complications. The number of needle repositioning attempts correlated significantly with puncture duration (Spearman r = 0.34, *p* < 0.0001), procedure duration (r = 0.34, *p* < 0.0001), and radiation exposure parameters including CTDIvol (r = 0.16, *p* = 0.0008), DLP (r = 0.21, *p* < 0.0001), and effective dose (r = 0.23, *p* < 0.0001), indicating increased complexity and resource utilization in procedures requiring multiple needle adjustments.

### Patient Positioning

In the prone position, 97.7% (43/44) of procedures were successful. In the left lateral position 97.9% (228/233) and in the right lateral position 98.2% (162/165) were successful. In addition, the technical success rate was 100% (5/5) in interventions in a combined right lateral/prone position and 100% (1/1) in a left lateral/prone position. The term ‘combined position’ refers to an oblique patient positioning between lateral decubitus and prone, typically achieved by placing cushions or bolsters under the flank to slightly rotate the patient. No repositioning occurred during the intervention. Detailed results are presented in Table 5. Patient positioning (prone, left lateral, right lateral, or combined) had no significant effect on technical success rates (*p* > 0.999). While patient positioning had no significant effect on technical success, it was significantly associated with puncture duration (*p* = 0.005). Median puncture times were 10.8 min (IQR 7.7–15.1) in right lateral, 7.3 min (IQR 6.2–8.8) in combined positions, 11.2 min (IQR 7.5–16.9) in left lateral, and 11.9 min (IQR 10.2–23.9) in prone position, with post-hoc analysis showing a significantly shorter duration in combined vs. prone positioning (*p* = 0.0044). Patient positioning (prone, left lateral, right lateral, or combined) had no significant effect on the number of needle repositioning attempts (Kruskal-Wallis *p* = 0.178; Dunn’s multiple comparisons: all adjusted *p* > 0.23).

**Table 5:** Technical success rates by patient positioning.

Patient positioning	Number of procedures	Number of successful procedures	Success rate (%)
Prone position	44 (9.6%)	43	97.7
Left lateral position	233 (50.9%)	228	97.9
Right lateral position	165 (36.0%)	162	98.2
Right lateral/prone position	15 (3.3%)	15	100
Left lateral/prone position	1 (0.2%)	1	100

### Puncture Levels

Puncture level selection was based on individual anatomy and any pre-existing surgical conditions, such as dorsal spondylodesis. A total of 458 puncture attempts were performed. The most frequently chosen puncture levels were L5/S1 (49.1%, 225/225; technical success rate: 100%), L4/5 (38.6%, 175/177; 98.9%), and L3/4 (3.3%, 14/15; 93.3%). Pairwise comparison revealed a significantly higher technical success rate for L5/S1 compared to L3/4 (*p* = 0.024), whereas no significant differences were observed between L5/S1 and L4/5 (*p* = 0.234) or between L4/5 and L3/4 (*p* = 0.265). Puncture duration did not differ significantly between the puncture levels L5/S1, L4/5, and L3/4 (Kruskal-Wallis test, *p* = 0.705; all post-hoc comparisons non-significant).

### Procedure and Puncture Duration

**Fig. 4:**
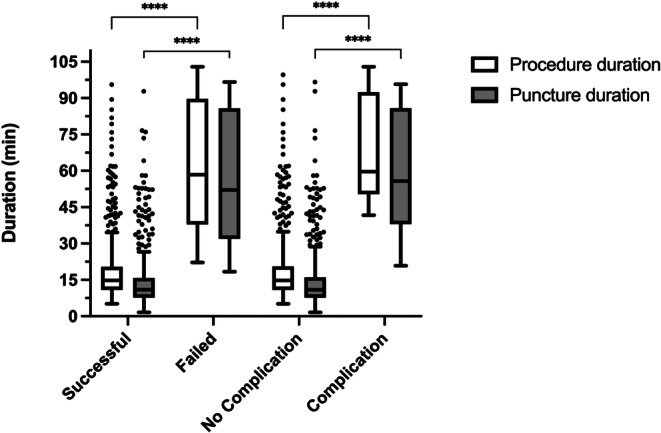
Duration of procedure and puncture by technical outcome and complication status. A small number of outliers were excluded from the graph to improve visualization; however, all data points were included in the statistical calculations. **** = *p* < 0.0001

The median puncture duration for all procedures was 11.1 min (IQR 7.7–16.2 min, range 1.6–96.6 min), and the median total procedure duration was 14.8 min (IQR 10.9–20.8 min, range 5.1–134.1 min). In patients with dorsal spondylodesis, median puncture duration was 11.9 min (IQR 9.1–17.6 min, range: 2.8–96.6 min) and the procedure duration was 16.3 min (IQR 12.5–22.1 min, range: 7.4–102.9 min). In patients without dorsal spondylodesis, these durations were 10.3 min (IQR 7.2–15.3, range: 1.6–76.0 min) and 14.0 min (IQR 10.4–20.6, range: 5.1–134.1 min), respectively. These differences were statistically significant (puncture duration: *p* = 0.0031; procedure duration: *p* = 0.0028), indicating greater procedural complexity in the spinal fusion group. In interventions with complications, the median puncture duration was 55.8 min (IQR 55.1–76, range: 20.9–95.7 min) and the median procedure duration was 59.7 min (IQR 59.1–81.9, range: 41.7–102.9 min). These differences were statistically significant for both puncture and procedure duration (*p* < 0.0001). In 8 of the 9 technically unsuccessful procedures with complete documentation, the median puncture duration was 52.2 min (IQR: 33.4–65.8; range: 18.4–96.6), and the median procedure duration was 58.4 min (IQR: 45.9–69.7; range: 22.2–102.9), both significantly longer than in successful interventions (puncture: 11.1 min; procedure: 14.8 min; *p* < 0.0001). Figure 4 illustrates the duration of procedure and puncture by technical outcome and complication status.

### Radiation Exposure

**Fig. 5:**
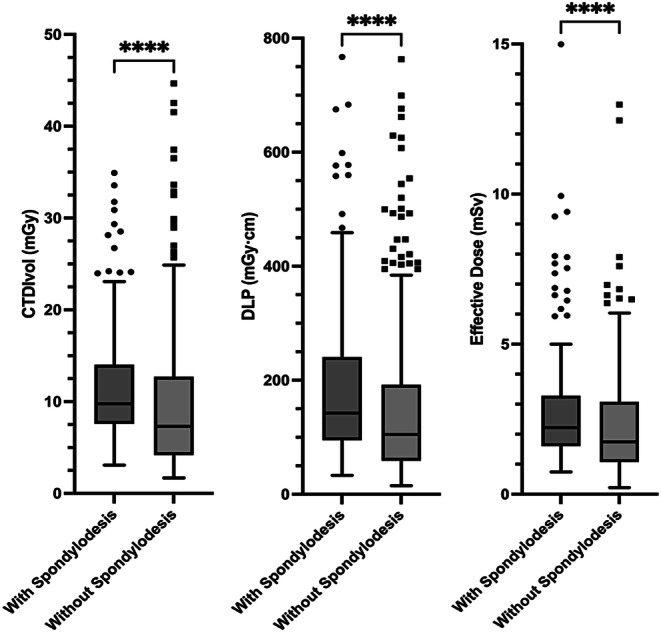
Radiation exposure parameters in patients with and without dorsal spinal fusion. A small number of outliers were excluded from the graphs to improve visualization; however, all data points were included in the statistical calculations. **** = *p* < 0.0001

The median CTDIvol of the 447 analyzed procedures was 8.67 mGy (IQR: 5.76–13.62), the median DLP was 121 mGy·cm (IQR: 75–218), and the median effective dose was 2.02 mSv (IQR: 1.35–3.17, range: 0.22–19.95). In patients with dorsal spondylodesis, the CTDIvol was 9.68 mGy (IQR: 7.53–13.92), the DLP was 142 mGy·cm (IQR: 95–240) and the effective dose was 2.22 mSv (IQR: 1.60–3.25, range 0.74–19.95). In patients without dorsal spondylodesis, lower values were recorded: CTDIvol 7.24 mGy (IQR: 4.12–12.64), DLP 105 mGy·cm (IQR: 59–191) and effective dose 1.74 mSv (IQR: 1.08–3.04, range 0.22–18.31). All three radiation dose parameters were significantly higher in patients with dorsal spondylodesis: CTDIvol (median 9.68 mGy vs. 7.24 mGy), DLP (142 vs. 105 mGy·cm), and effective dose (2.22 vs. 1.74 mSv; all *p* < 0.0001, Fig. 5). These differences underline the increased technical demands and prolonged scan durations in this patient subgroup. In procedures with complications, radiation exposure was higher: CTDIvol 9.78 mGy (IQR: 5.08–22.50), DLP 414 mGy·cm (IQR: 212–447) and effective dose 3.82 mSv (IQR: 3.45–4.25, range 1.61–19.95). Although CTDIvol did not differ significantly between procedures with and without complications (median 9.78 mGy vs. 8.67 mGy; *p* = 0.608), both DLP (414.4 vs. 121.0 mGy·cm; *p* = 0.040) and effective dose (3.82 vs. 2.01 mSv; *p* = 0.030) were significantly higher in procedures with complications, likely reflecting the need for additional scan acquisitions in technically demanding cases. In unsuccessful procedures, radiation exposure was highest: CTDIvol 17.8 mGy (IQR: 8.58–30.87), DLP 414 mGy·cm (IQR: 119–599), and effective dose 4.25 mSv (IQR: 2.54–7.69, range 1.61–12.45); all values were significantly higher compared to successful procedures (CTDIvol 17.8 mGy vs. 8.7 mGy, *p* = 0.047; DLP 414 mGy·cm vs. 121 mGy·cm, *p* = 0.014; effective dose 4.25 mSv vs. 2.02 mSv, *p* = 0.002). A summary of radiation exposure parameters across patient subgroups is provided in Table 6. Radiation dose parameters varied significantly by patient positioning. Median CTDIvol was highest in the prone position (11.52 mGy), followed by right lateral (8.97 mGy), left lateral (8.35 mGy), and lowest in the combined position (4.17 mGy; *p* < 0.0001). DLP showed a similar pattern, with medians of 168.9 mGy·cm (prone), 112.5 mGy·cm (right lateral), 127.9 mGy·cm (left lateral), and 51.4 mGy·cm (*p* = 0.0001). Effective dose was also highest in prone position (2.45 mSv) and lowest in combined position (1.17 mSv; *p* = 0.0143). Post-hoc analysis revealed that the combined position consistently yielded significantly lower radiation doses compared to other positions, whereas no significant differences were found between prone, left lateral, and right lateral positions.

**Table 6:** Radiation exposure.

Parameters	Total procedures	Procedures with dorsal spondylodesis	Procedures without dorsal spondylodesis	Unsuccessful procedures	Interventions with complications
CTDIvol (median, IQR)	8.67 (5.76–13.62)	9.68 (7.53–13.92)	7.24 (4.12–12.64)	17.80 (8.58–30.87)	9.78 (5.08–22.50)
DLP (median, IQR)	120.99 (74.73–218.36)	141.75 (94.81–239.92)	104.66 (58.66–191.14)	414.38 (119.27–598.70)	414.38 (211.65–446.61)
Effective dose (median, IQR, range)	2.02 (1.35–3.17, 0.22–19.95)	2.22 (1.60–3.25, 0.74–19.95)	1.74 (1.08–3.04, 0.22–18.31)	4.25 (2.54–7.69, 1.61–12.45)	3.82 (3.45–4.25, 1.61–19.95)

*CTDI* Computed Tomography Dose Index, *DLP* Dose-Length Product

## Discussion

This study provides a comprehensive evaluation of CT-guided intrathecal nusinersen administration in adolescent and adult patients with SMA in a routine clinical setting. With a high technical success rate of 98.3%, it confirms the feasibility and reliability of CT-guided lumbar puncture—even under complex anatomical conditions such as dorsal spinal fusion [19, 20].

The observed differences in technical success, duration, and radiation exposure between patients with and without spinal instrumentation reflect the increased technical demands in surgically altered anatomy, which was also evident in the higher repositioning rates observed in this subgroup [21, 22]. In patients with dorsal spondylodesis, higher radiation doses are likely attributable to a combination of two factors: (1) automatic dose modulation caused by metallic implants and beam hardening artifacts, which increase tube output to maintain image quality, and (2) prolonged scan times and repeated acquisitions resulting from greater procedural complexity and more frequent needle repositioning. The latter is supported by the significant correlation between procedure duration and dose parameters observed in our study. These findings suggest that increased procedural complexity—due to anatomical challenges or difficult access—was associated with a higher need for repositioning attempts. In our cohort, the median number of needle repositioning attempts was 0 (range 0–5), yet repositioning occurred significantly more often in patients with dorsal spondylodesis (*p* = 0.012), reflecting increased procedural complexity in this subgroup. These findings are consistent with previous reports [23], which also demonstrated higher effective doses in patients with dorsal spondylodesis compared to those without. Our study extends these observations by confirming them in a substantially larger cohort and by providing a more detailed analysis of procedural duration and technical outcomes. Although patients with dorsal spondylodesis demonstrated a slightly lower technical success rate and significantly longer procedure and puncture durations, the outcomes remained favorable overall. Nonetheless, the technical success rate of over 97% in the fusion subgroup underlines the robustness of the CT-guided approach. These findings highlight that even challenging cases can be managed safely and effectively with proper planning and technique [24].

Importantly, procedures associated with complications or technical failure showed significantly prolonged durations and markedly increased radiation exposure. In our series, most failed interventions were attributable to an inability to access the intrathecal space due to complex spinal anatomy, including extensive dorsal instrumentation and severe scoliosis. In one case, the procedure was discontinued prematurely because of pain intolerance reported by the patient. These findings underscore the importance of minimizing procedure complexity through individualized preparation and image-based planning, particularly in patients with altered spinal anatomy or previous surgical interventions [21].

The low overall complication rate of 1.1%, with no long-term sequelae, further supports the safety of this method and is consistent with previously published data [25–27]. The predominance of complications in the fusion group is noteworthy and may serve as a reminder for heightened procedural awareness in this subgroup. Importantly, both failed and complicated procedures were associated with longer durations and markedly increased radiation doses—underscoring the importance of careful planning, patient-specific protocol optimization, and dose-saving strategies.

Patient positioning did not appear to have a substantial impact on technical success rates. This suggests that procedural positioning can be tailored flexibly based on anatomical and clinical considerations, without compromising safety or effectiveness. The oblique alignment may facilitate access to interlaminar spaces, especially in patients with dorsal instrumentation, by reducing the overlap of metallic hardware and widening the approach angle. These factors may explain the observed shorter puncture times and lower radiation exposure compared to the fully prone position.

The puncture level L5/S1 proved to be the most accessible and reliable, achieving a 100% success rate. In contrast, alternative or repeated punctures at other levels were more commonly associated with technical failure, particularly in the presence of prior instrumentation.

Compared to alternative imaging modalities such as ultrasound or fluoroscopy, CT-guided administration offers superior anatomical resolution and planning flexibility—at the expense of ionizing radiation. Previous studies have shown that fluoroscopic guidance is a safe and effective approach for intrathecal administration of nusinersen, even in patients with complex SMA anatomy, achieving high technical success and low complication rates [28]. In adult SMA cohorts including cases with spinal fusion, both fluoroscopy- and CT-guided punctures demonstrated 100% success rates, with fluoroscopy being primarily used for posterior interlaminar access and CT reserved for transforaminal or surgically modified approaches [29]. These findings support a tailored approach, in which fluoroscopy serves as the first-line modality in straightforward anatomy, while CT guidance remains indispensable in anatomically complex or instrumented spines. Our results suggest that in complex anatomical cases, the benefits of CT outweigh the risks, particularly when dose-optimized protocols are in place. In straightforward cases or in younger patients, sonographic or fluoroscopic approaches may be preferable to minimize radiation burden, particularly in the context of long-term therapy requiring usually three annual injections, where cumulative exposure and adherence to the as low as reasonably achievable (ALARA) principle become clinically relevant [30, 31]. Although median effective doses per procedure remained moderate (~2 mSv), consistent with values reported in previous studies, cumulative exposure may become clinically significant, especially in younger patients [32]. Therefore, dose optimization strategies should be integral to protocol design, such as minimizing scan length, using low-dose settings, and leveraging iterative reconstruction [21].

A key methodological strength of this study lies in the standardized documentation of procedural parameters and radiation exposure across a large number of procedures. Nevertheless, several limitations must be acknowledged. The retrospective design precludes causal inference and is susceptible to documentation bias. Additionally, the lack of a control group with other imaging techniques (e.g., sonographically or fluoroscopically guided punctures) limits the generalizability of findings [33–35]. Future prospective studies comparing CT guidance to alternative imaging modalities in anatomically challenging cohorts are warranted to further refine selection criteria and establish procedural algorithms. BMI data were not consistently documented in the retrospective dataset and could therefore not be analyzed, which may have influenced procedure duration and radiation exposure.

## Conclusion

CT-guided intrathecal administration of nusinersen is a safe, effective and well-established technique for treating patients with SMA, including those with complex spinal anatomy such as dorsal spondylodesis. The high technical success rate, low complication rate, and moderate radiation exposure support the clinical utility of this approach in routine practice. Individualized selection of puncture level and patient positioning, tailored to anatomical conditions, enables reliable implementation even in challenging cases. These findings highlight the importance of structured procedural planning and underscore the value of CT guidance as a robust imaging modality for long-term SMA therapy. While CT guidance ensures high reliability in complex anatomy, fluoroscopy may be preferable in straightforward cases with lower radiation exposure; prospective studies are needed to define optimal modality selection in SMA.
